# Difference‐Makers for Collecting Sexual Orientation and Gender Identity Data in Oncology Settings

**DOI:** 10.1002/cam4.70727

**Published:** 2025-03-06

**Authors:** Mandi L. Pratt‐Chapman, Edward J. Miech, Megan A. Mullins, Shine Chang, Gwendolyn P. Quinn, Shail Maingi, Matthew B. Schabath, Charles Kamen

**Affiliations:** ^1^ Department of Medicine, GW Cancer Center The George Washington University School of Medicine and Health Sciences Washington DC USA; ^2^ Department of Emergency Medicine Indiana University School of Medicine Indianapolis Indiana USA; ^3^ O'Donnell School of Public Health, Simmons Comprehensive Cancer Center UT Southwestern Medical Center Dallas Texas USA; ^4^ Division of Cancer Prevention and Population Sciences, Department of Epidemiology The University of Texas MD Anderson Cancer Center Houston Texas USA; ^5^ Departments of OB‐GYN, Population Health, Perlmutter Cancer Center Grossman School of Medicine, New York University New York City New York USA; ^6^ Dana‐Farber Cancer Institute Cancer Care Equity Program Boston Massachusetts USA; ^7^ Department of Cancer Epidemiology H. Lee Moffitt Cancer Center & Research Institute Tampa Florida USA; ^8^ University of Rochester Medical Center Rochester New York USA

**Keywords:** coincidence analysis, gender identity, implementation, oncology, sexual orientation

## Abstract

**Purpose:**

The purpose of this analysis was to identify key difference‐making conditions that distinguish oncology institutions that collect sexual orientation and gender identity (SOGI) data across a sample of American Society of Clinical Oncology (ASCO) members.

**Methods:**

From October to November 2020, an anonymous 54‐item web‐based survey was distributed to ASCO members. Coincidence analysis was used to identify difference‐making conditions for the collection of SOGI data.

**Results:**

ASCO members' responses to just three items consistently distinguished practices that reported collecting both SO and GI data (*n* = 25) from those who did not (*n* = 20): (1).“Do you ask your patients what pronouns they want you to use for them?”; (2) “Institutional leadership supports collecting SOGI data from patients”; and (3)“Does the electronic health record (EHR) at your institution have a specific section to collect information about patients' SOGI?” The positive model exhibited both reliability (consistency = 0.87, or 20/23) and explanatory breadth (coverage = 0.80, or 20/25). The negative model for SOGI data collection consisted of different responses to the same three items and likewise showed both reliability (consistency = 0.94, or 16/17) and explanatory breadth (coverage = 0.80, or 16/20).

**Conclusions:**

Specific levels of leadership support, frequency of asking patients about pronouns, and the presence or absence of EHR record structure were difference‐makers for collecting SOGI data in this sample. The study underscores the importance of leadership support, structured data fields, and attention to patient pronouns, which are aligned with the ASCO and National Institutes of Health calls to action.

## 1 Introduction

Lesbian, gay, bisexual, transgender, queer, intersex persons and other individuals who do not identify as heterosexual and cisgender (LGBTQI+) experience health disparities across the cancer continuum due to complex factors. For example, LGBTQI+ people have a higher prevalence of alcohol and tobacco consumption due to a long history of targeted marketing [1]. Lack of clinical guidelines specific to gender‐diverse people and gender dysphoria results in lower rates of screening for some cancers among transgender persons [2]. LGBTQI+ people may have different preferences for care, post‐surgical reconstruction, and tailored support services that are often not available. Attempts to address these disparities have been hampered by a lack of consistent identification of LGBTQI+ patients in cancer prevention, detection, treatment, and survivorship services, as well as in cancer registries and surveillance databases [2].

The American Society of Clinical Oncology's (ASCO) landmark 2017 position statement highlighted the critical need to improve the collection of sexual orientation and gender identity (SOGI) data to identify cancer‐related disparities affecting LGBTQI+ people [3]. In 2024, the American Cancer Society issued a report highlighting disparities in cancer among LGBTQI+ people and called out the critical need to routinely collect SOGI data to better identify and address their needs [2]. Collection of SOGI data remains unsystematic despite this call and similar calls from national organizations including the United States Preventive Services Task Force and despite studies demonstrating the widespread acceptability of SOGI questions among both LGBTQI+ patients and cisgender/heterosexual patients [4, 5, 6].

Previously identified factors associated with a greater likelihood of SOGI data collection in oncology practice have included: (1) leadership support for the collection of these data, (2) resources for data collection, (3) understanding of the importance of SOGI data collection to oncology clinical practice, and (4) the geographic context of the practice (e.g., location of practice in the Northeastern part of the U.S.) [7, 8]. In this analysis, we conducted a secondary analysis of data from a national survey of ASCO members to identify key difference‐makers associated with the collection of SOGI data in oncology practices using a novel method: coincidence analysis (CNA) [7].

## 2 Methods

### 2.1 Ethical Review

Per ASCO internal policies, this study was considered health care operations quality improvement. Thus, no institutional review board approval was sought. Informed consent was assumed if the respondent completed the survey in REDCap.

### 2.2 Study Population

We conducted a survey (S1) of oncology professionals in the U.S. to explore SOGI data collection practices. The recruitment approach for the larger sample (*n* = 257) is described elsewhere and included direct recruitment of ASCO members from ASCO membership lists, as well as snowball sampling of other oncology professionals, including physicians, nurses, and patient navigators [7]. The results presented here are from a secondary analysis of the 45 participants who identified as ASCO members in their response to the survey. While our prior analysis included a more heterogeneous sample of individuals by discipline, we sought to restrict this analysis to ASCO members to better understand perspectives at the level of physicians. We selected ASCO members given ASCO's landmark 2017 statement advocating for oncologists in general (and ASCO members specifically) to collect SOGI data [3].

### 2.3 Data Collection

The 54‐item survey was created by the SGM Task Force of the ASCO Health Equity Committee and is more fully described elsewhere [7]. The survey included questions from a prior validated instrument as well as de novo items asking respondents about institutional policies and practices regarding SOGI data collection; respondent knowledge, beliefs, attitudes, and individual practices regarding SOGI data collection; and respondent perception of the importance of SOGI data to clinical practice [9]. Fifteen items queried demographic items such as professional role, including involvement in clinical care, research, or both; institution type; sexual orientation; gender identity; age; race; ethnicity; political views; LGBTQ+ family members; LGBTQ+ friends and/or coworkers; and LGBTQ+‐specific training.

### 2.4 Data Analysis

Coincidence Analysis (CNA) was utilized to identify the key difference‐makers for collecting SOGI data among ASCO members. With over 100 publications in the peer‐reviewed literature, CNA is an emerging analytical approach within the larger family of methods collectively known as configurational analysis. CNA is a case‐based approach to data analysis that uses Boolean algebra, set theory, and formal logic to determine crucial conditions that consistently and uniquely yield an outcome of interest [10, 11, 12].

CNA allowed us to identify both the (a) specific items and the (b) particular response levels for those items that distinguished oncology settings that collected both SO and GI data from their patients from settings that did not. CNA has the capacity to handle real‐world complexity through its ability to model both conjuncts and disjuncts. Conjuncts are when specific conditions together explain an outcome and use the Boolean operator “AND”; disjuncts are when multiple pathways lead to the same outcome and use the Boolean operator “OR.” CNA's capability to model conjuncts and disjuncts, along with its versatility related to small sample sizes, made this approach well suited for this analysis. While regression analysis models relationships between variables, CNA analysis identifies the key difference‐makers that account for the presence or absence of a desired outcome. Drawing upon a different kind of math as well as a different analytic target, findings from CNA offer a different set of insights into complex phenomena that can complement results generated through traditional regression methods [13, 14].

R, the R package “cna” for Coincidence Analysis, RStudio, and Microsoft Excel were used to support this analysis [15].

### 2.5 Data Preparation

As the CNA analysis requires the dependent variable to be expressed as a categorical factor, a dichotomized metafactor was created from the SOGI outcome and coded as “1” when a site reported collecting both SO and GI information from their patients and “0” when they did not. To achieve data reduction, we used a configurational approach using the “minimally sufficient conditions” (i.e., “msc”) function, a routine that has been described in prior research [9, 10, 11]. To summarize, the “msc” function was applied within the R package “cna” to look across all 45 oncology settings and all 54 items at once in the original dataset to identify combinations or “bundles” of specific items at particular response levels that had especially strong connections to both the presence and the absence of the SO and GI data collection outcome.

CNA applies a mathematical algorithm to remove extraneous, superfluous, or redundant factors from final models, leaving only key difference‐making conditions that distinguish cases with or without an outcome. The Boolean difference‐making configurations identified by CNA thus comprise a “minimal theory” that explains the presence or absence of an outcome. This exhaustive process considered every possible 1‐, 2‐, and 3‐condition combination of values within the original dataset and identified all bundles that met the specified consistency threshold. During this exploratory data analysis, the msc function was run at five different consistency levels: 95%, 90%, 85%, 80%, and 75% [9, 10, 11]. The exploratory data analysis was limited to bundles of 3 conditions because exhaustively searching for every possible combination of 4 or more conditions proves computationally unwieldy, and because the number of cases that meet a given consistency threshold while at the same time instantiating the intersection of 4 or more specific factor values is generally very small.

When reviewing this mathematical output, we focused on configurations with “best of class” coverage scores (i.e., top coverage score among bundles with the same number of conditions); with items where the same question (at different response levels) linked directly to both the presence and absence of the outcome; and were consistent with logic, theory and prior knowledge. Using this approach, we inductively analyzed the entire dataset and used the output to identify a subset of items for model development during the next step of the CNA analysis.

### 2.6 Model Development

During model development, the goal was to develop a model with acceptable levels of consistency and coverage. Consistency is a measure of reliability, indicating how often cases identified by the model also have the outcome present. Coverage is a measure of explanatory breadth, indicating how many cases that have the outcome present are covered by the model. Our goal was to identify an overall model that met all of the following criteria: ≥ 80% scores for both consistency and coverage; aligned with theory and background knowledge; and no model ambiguity (i.e., there was only one solution) [12]. At this step of the analysis, any cases with missing values for any factors used in preliminary model development were dropped, a standard procedure within configurational analysis.

## 3 Results

This study included 45 ASCO members (Table 1) who work at university hospitals (60%), community/private hospitals (22.2%), private practices (11.1%), and other institutions (6.7%). Nearly all (95.6%) of these 45 respondents were physicians, including 40 oncologists, 2 surgeons, and a hematologist, along with a PhD‐level cancer geneticist and a physician's assistant. These individuals were predominantly heterosexual (91.1%) and were roughly equivalent in terms of reported sex: male (44.4%) and female (48.9%). Most (66.7%) respondents were White (13.3% Asian, 6.7% Black, 8.9% other, 4.4% did not disclose).

**TABLE 1:** American society of clinical oncology member participant demographics.

	*N* (%)
Age	
< 45	10 (22.2)
45–60	19 (42.2)
60–74	14 (31.1)
Missing	2 (4.4)
Institution type	
University Hospital	27 (60.0)
Community/Private Hospital	10 (22.2)
Private practice	5 (11.1)
Other	3 (6.7)
Sexual orientation	
Gay	2 (4.4)
Heterosexual	41 (91.1)
Prefer not to answer	1 (2.2)
Queer	1 (2.2)
Intersex identity	
Yes	1 (2.2)
No	42 (93.3)
Prefer not to answer	2 (4.4)
Gender identity	
Female	22 (48.9)
Male	20 (44.4)
Non‐binary	1 (2.2)
Prefer not to answer	2 (4.4)
Transgender, gender diverse, and/or gender queer identity	
Yes	2 (4.4)
No	41 (91.1)
Prefer not to answer	2 (4.4)
Race	
Asian	6 (13.3)
Black	3 (6.7)
White	30 (66.7)
Other	4 (8.9)
Prefer not to answer	2 (4.4)
Ethnicity	
Hispanic/latino	1 (2.2)
Not hispanic/latino	41 (91.1)
Prefer not to answer	2 (4.4)
Education	
Doctoral degree	44 (97.8)
Other professional degree	1 (2.2)

Three factors consistently distinguished respondents who reported collecting both SO and GI data (*n* = 25) from those who did not (*n* = 20).

### 3.1 Positive Model

**FIGURE 1:**
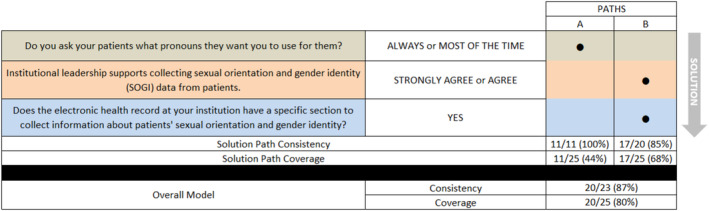
Conditions directly linked to collection of sexual orientation and gender identity data (positive model).

The positive model for SOGI data collection consisted of two solution pathways: replying “always” or “most of the time” to the question “Do you ask your patients what pronouns they want you to use for them?” OR replying “strongly agree” or “agree” to the statement “Institutional leadership supports collecting sexual orientation and gender identity (SOGI) data from patients” AND replying “yes, the health record has a specific page/tab/section for SOGI data” to the question “Does the electronic health record at your institution have a specific section to collect information about patients' sexual orientation and gender identity?” As shown in Figure 1, the positive model demonstrated both reliability (consistency = 0.87, or 20/23) and explanatory breadth (coverage = 0.80, or 20/25). In other words, 20 of the 23 cases indicated by the positive model correctly identified cases with the outcome of interest (e.g., SOGI data are collected systematically), whereas 20 of the 25 cases with the outcome of interest were covered by the positive model [16].

Figure 1 shows the two solution pathways that led to the outcome of collecting both SO and GI. Of the 25 sites that indicated they collected SOGI, 11 said they always (*n* = 6) or most of the time (*n* = 5) asked patients their pronouns. Seventeen sites agreed (*n* = 2) or strongly agreed (*n* = 15) they had institutional leadership support to collect SOGI; and of these, nine reported having both a structured field in the EHR and leadership support. The two respondents from private practice that collected SOGI were covered by both pathways: Both reported always or most of the time asking patients about their pronouns; both strongly agreed that they had institutional leadership support to collect SOGI; and both reported that a structured field existed in the EHR. Of the 18 members from university sites that reported collecting SOGI, 7 reported always (*n* = 4) or most of the time (*n* = 3) asking patients about their pronouns. Fourteen of these members strongly agreed (*n* = 11) or agreed (*n* = 3) that they had institutional leadership support to collect SOGI, and 10 of these reported that they both had institutional leadership support and a structured field in the EHR.

### 3.2 Negative Model

**FIGURE 2:**
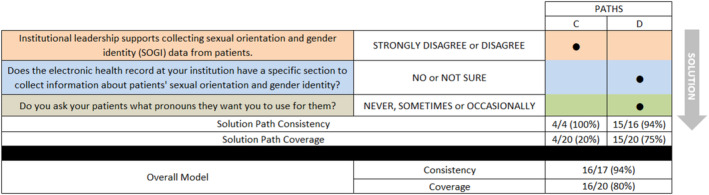
Conditions directly linked to non‐collection of sexual orientation and gender identity data (negative model).

The negative model for SOGI data collection (i.e., for the absence of the outcome) consisted of two solution pathways: replying “strongly disagree” or “disagree” that leadership supports collecting SOGI data OR “giving any response other than” yes, “the health record has a specific page/tab/section for SOGI data” to the question “Does the electronic health record at your institution have a specific section to collect information about patients' sexual orientation and gender identity?” AND replying “sometimes,” “occasionally” or “never” to how often they ask patients about pronouns. As shown in Figure 2, the negative model showed both reliability (consistency = 0.94, or 16/17) and explanatory breadth (coverage = 0.80, or 16/20). In other words, 16 of the 17 cases indicated by the negative model correctly identified cases with the outcome of interest (i.e., in this case, the absence of SOGI data collection), whereas 16 of the 20 cases with the outcome of interest were covered by the negative model.

Figure 2 shows the two solution pathways leading to the negative outcome of not collecting both SO and GI. Of the 20 sites that indicated they did not collect SOGI, they varied in asking about pronouns: never (*n* = 11), sometimes (*n* = 5), and occasionally (*n* = 4). In terms of leadership support for data collection, there was great variability: strongly agree (*n* = 1), agree (*n* = 5), no opinion (*n* = 10), disagree (*n* = 3), strongly disagree (*n* = 1). Of these sites that did not collect SOGI, five indicated that the EHR had structured fields to do so while the rest reported not having structured fields (*n* = 8) or not being sure (*n* = 7).

## 4 Discussion

While heralded as critical by ASCO and other professional bodies and required by the Centers for Medicare and Medicaid under Meaningful Use standards since 2015, the need to collect SOGI data in oncology practice is not universally understood [3, 17, 18, 19]. Relevant to cancer risk, sexual and gender minorities have historically experienced intense marketing from tobacco and alcohol corporations. As a result, SGM people have a higher prevalence of risk factors for certain cancers associated with alcohol and tobacco [20]. Lack of inclusion of sexual orientation and gender identity data in clinical trials critically limits the data needed for providing tailored screening and treatment guidelines for LGBTQI+ people [21]. Differences in endogenous and exogenous hormone balance and anatomy have major implications for breast and prostate cancer screening recommendations and treatment. While the American College of Radiology has provided vignettes to guide breast cancer screening for transgender individuals, systematic data collection of sex assigned at birth, gender identity, and organs present would facilitate the refinement of cancer screening guidelines and ensure more comprehensive reach to those at greater risk [22].

Further, LGBTQI+ people have unique treatment and supportive care needs due to discrimination and denial of healthcare; obtrusive clinical interactions; and lack of research to provide tailored supportive care options to same‐sex couples. Trauma‐informed screening approaches are critical: transgender men often face extreme gender dysphoria for cervical cancer screening, and intersex individuals may have undergone numerous clinical encounters without consent. Supportive care has historically been centered on cisgender, heterosexual people. Understanding the specific needs of LGBTQI people when addressing sexual health sequelae from cancer treatment as well as psychosocial support needs affects the degree to which quality care is available and offered to LGBTQI people affected by cancer [23].

This secondary analysis found that the presence of leadership support to collect SOGI data, a place in the EHR to store SOGI data, and clinicians routinely asking about patients' pronouns were associated with the collection of SOGI data in oncology practice. Conversely, the lack of leadership support for SOGI data collection, the lack of known EHR fields for SOGI data, and the absence of asking patients about pronouns was associated with no SOGI data collection. Given that positive and negative models are constructed independently in CNA, the convergence of the models in this analysis adds further emphasis to the importance of these three factors.

This analysis complements our initial analysis that used traditional statistical methods, which found significant associations of SOGI data collection with leadership support, resources for data collection, and understanding of why SOGI data are important to oncology practice [7]. The present analysis provides additional clarity concerning the types of resources that are important (e.g., an EHR with known structured fields for SOGI data) and what conditions might be present (e.g., asking about pronouns) in settings that actually collect SOGI data in clinical practice. These findings are consistent with those identified in other health care settings such as cardiology and pediatrics [24, 25].

The Centers for Medicaid and Medicare services recently offered recommendations, guidance, and suggested wording for the collection of SOGI data, noting the need for such data to better understand health care disparities. Additionally, other commentaries and empirical data have noted the need for continuous collection of SOGI data as sexual orientation and gender identity status are fluid and may change over time [26, 27, 28]. The findings of this study add to the ongoing dialogue around necessary and sufficient factors for the collection of SOGI data, and how SOGI data collection may conversely influence practice settings.

For oncology leaders who wish to champion SOGI data collection, this study provides support for strengthening leadership commitment to comprehensive, accurate data collection. While structured fields for SOGI are required by the Centers for Medicare and Medicaid under Meaningful Use Standards, not all clinicians and staff know these fields are available, where to find them, how to collect data appropriately, and how to use data to inform care management. Training to address these knowledge gaps is critically needed. Given the current sociopolitical landscape in the U.S. that has witnessed a backlash in the safety of LGBTQI+ people, the authors emphasize that institutions should systematically collect SOGI data to guide clinical and supportive care and improve the future evidence base for guideline development; however, individuals should not be required to document these data in their medical records if they prefer not to or have safety concerns.

### 4.1 Limitations

In terms of limitations, this study was based on a convenience sample, and biases in sampling may have existed. In the larger survey sample, a higher number of respondents identified as sexual and gender minorities than the national average, indicating a potential bias toward positive beliefs about the importance of SOGI data collection. In addition, the sub‐sample used for this analysis was small (*n* = 45), though sufficient for the analytic technique employed. Finally, difference‐making factors are not inherently causal, and additional work is required (e.g., independent replication, randomized trials) to determine the strength and direction of any causal relationships. For example, the authors interpret the behavior of asking about pronouns to be an effect of the larger institutionalization of asking about SOGI rather than a predictor of SOGI data collection. We also did not collect data about geography, and therefore could not examine potential differences in practice by geographic location.

The results of this analysis should be treated as providing novel findings about difference‐making factors for SOGI collection that can both inform and complement findings generated through other approaches, including statistical analysis and qualitative research, with the goal of implementing strategies to bolster widespread SOGI data collection in oncology.

## 5 Conclusions

Leadership support, knowing where to find structured SOGI fields in the EHR, and asking patients about pronouns consistently distinguished ASCO members' practices where SOGI data was systematically collected from those where it was not. The study supports the need for leadership to set standards for SOGI data collection and ensure that structured data fields are operational in EHRs, and clinicians know where to find those fields to support data collection. Based on our findings, providing education for the oncology workforce on why collecting SOGI data is important, how to respectfully inquire about pronouns, and where to input and locate SOGI variables in EHR systems are high‐yield initiatives that can significantly bolster SOGI data collection. Leadership commitment to improve SOGI data collection will be critical to building evidence for improved guideline development and personalized oncology care to meet the needs of LGBTQI+ persons.

## Author Contributions

**Mandi L. Pratt‐Chapman:** conceptualization (equal), funding acquisition (lead), investigation (lead), project administration (lead), supervision (lead), writing – original draft (equal), writing – review and editing (lead). **Edward J. Miech:** data curation (lead), formal analysis (lead), methodology (lead), software (lead), writing – original draft (equal), writing – review and editing (supporting). **Megan A. Mullins:** writing – review and editing (supporting). **Shine Chang:** writing – review and editing (supporting). **Gwendolyn P. Quinn:** writing – review and editing (supporting). **Shail Maingi:** writing – review and editing (supporting). **Matthew B. Schabath:** writing – review and editing (supporting). **Charles Kamen:** funding acquisition (supporting), investigation (supporting), writing – review and editing (supporting).

## Ethics Statement

Per the protocols of the American Society of Clinical Oncology, this study was considered a quality improvement and was not submitted to an IRB.

## Consent

The authors have nothing to report.

## Conflicts of Interest

The authors declare no conflicts of interest.

## Supporting information


Data S1.

## Data Availability

Data will be made available to academic researchers wishing to use data for non‐commercial purposes upon the request of the corresponding author.
