# Supplementary material for: Difference‐Makers for Collecting Sexual Orientation and Gender Identity Data in Oncology Settings
Source: Cancer Med. 2025 Mar 6;14(5):e70727. doi: 10.1002/cam4.70727 (PMC11883291; doi:10.1002/cam4.70727)
Supplement: Supplementary file 1 — Data S1. [file CAM4-14-e70727-s001.docx]

ASCO Research: DATA FOR QUALITY ONCOLOGY CARE

| **QUESTIONS ABOUT PRACTICES: Thinking about the institutions at which you work, please answer the following questions.** | | | | | |
| --- | --- | --- | --- | --- | --- |
| What is your role at your institution? | - Medical Oncologist - Radiation Oncologist - Surgeon - Nurse - Nurse Practitioner - Physician's Assistant - Researcher/Scientist/Academic - Administrator - Other: ______________ | | | | |
| Please specify: | _________________________________________________________ | | | | |
| Do any of the institutions where you work provide direct patient care? | - Yes - No | | | | |
| Thinking about the patient care institution at which you spend the greatest amount of time, please answer the following questions. | | | | | |
| Does the institution where you work collect information about patients' sexual orientation in the health record (e.g., Straight or heterosexual; Lesbian, gay, or homosexual; Bisexual or other)? | - Yes - No - Not Sure | | | | |
| How consistently are data about sexual orientation collected in patient health records? | - Collected in all records - Collected in most records - Collected in some records - Collected in a few records - Not sure | | | | |
| Does the institution where you work collect information about patients' gender identity in the health record (e.g., Male; Female; Transgender, gender diverse, non-binary or other)? This would be distinct from or in addition to the standard male-female sex field. | - Yes - No - Not Sure | | | | |
| How consistently are data about gender identity collected in the health record? | - Collected in all records - Collected in most records - Collected in some records - Collected in a few records - Not sure | | | | |
| **Please respond to the following statements:** | | | | | |
|  | Strongly Agree | Agree | Disagree | Strongly Disagree | No Opinion |
| Institutional leadership supports collecting sexual orientation and gender identity (SOGI) data from patients. |  |  |  |  |  |
| My coworkers support collection of SOGI data from patients. |  |  |  |  |  |
| There are resources to support SOGI data collection at my institution. |  |  |  |  |  |
| I feel empowered to support SOGI data collection at my institution. |  |  |  |  |  |
| Does the institution where you work use an electronic health record? | - Yes - No - Not Sure | | | | |
| Does the electronic health record at your institution have a specific section to collect information about patients' sexual orientation and gender identity? | - Yes, the health record has a specific page/tab/section for SOGI data - No, the health record does not have a specific section, but it can be recorded as unstructured data in notes - No, the health record does not allow for collection of SOGI data - Not sure | | | | |
| Are you involved in providing direct patient care? | - Yes - No | | | | |
| **Please respond to the following statements:** | | | | | |
|  | Strongly Agree | Agree | Disagree | Strongly Disagree | No Opinion |
| Do you consider your patients' sexual orientation when discussing their care with them? |  |  |  |  |  |
| Do you consider your patients' gender identity when discussing their care with them? |  |  |  |  |  |
| Do you ask your patients what name they want you to use for them? |  |  |  |  |  |
| Do you ask your patients what pronouns they want you to use for them? |  |  |  |  |  |
| **Please respond to the following statements:** | | | | | |
|  | Strongly Agree | Agree | Disagree | Strongly Disagree | No Opinion |
| It is important to know the sexual orientation of my patients to provide the best care. |  |  |  |  |  |
| It is important to know the gender identity of my patients to provide the best care. |  |  |  |  |  |
| I am comfortable discussing my patients' sexual orientation. |  |  |  |  |  |
| I am comfortable discussing my patients' gender identity. |  |  |  |  |  |
| Most of my patients would be comfortable if they were asked about their sexual orientation. |  |  |  |  |  |
| Most of my patients would be comfortable if they were asked about their gender identity. |  |  |  |  |  |
| I am confident in my knowledge of the cancer care needs of transgender and gender diverse patients. |  |  |  |  |  |
| I am confident in my knowledge of the cancer care needs of lesbian patients. |  |  |  |  |  |
| I am confident in my knowledge of the cancer care needs of gay patients. |  |  |  |  |  |
| I am confident in my knowledge of the cancer care needs of bisexual patients. |  |  |  |  |  |
| I am confident in my knowledge of the cancer care needs of intersex patients. |  |  |  |  |  |
| I would like to be listed as an LGBTQI friendly provider. |  |  |  |  |  |
| Are you currently involved in conducting research? | - Yes - No | | | | |
| Do you collect information about sexual orientation as part of your research studies (e.g., Straight or heterosexual; Lesbian, gay, or homosexual; Bisexual or other)? | - Yes - No - Not sure - My research does not involve identifiable human subjects | | | | |
| Do you collect information about patients' gender identity as part of your research studies (e.g., Male; Female; Transgender, gender diverse, non-binary or other)? This would be distinct from or in addition to the standard male-female sex field. | - Yes - No - Not sure - My research does not involve identifiable human subjects | | | | |
|  | Strongly Agree | Agree | Disagree | Strongly Disagree | No Opinion |
| It is important to know the sexual orientation of human subjects in research studies. |  |  |  |  |  |
| It is important to know the gender identity of human subjects in research studies. |  |  |  |  |  |
| GENERAL QUESTIONS | | | | | |
| What barriers exist to collecting sexual orientation and gender identity data at your institution? Please list: | _________________________________________________________ | | | | |
| What would make it easier to collect sexual orientation and gender identity data at your institution? | _________________________________________________________ | | | | |
| DEMOGRAPHICS | | | | | |
| What is your gender identity? | - Female - Male - Non-binary - Identify as:   ______________________   - Prefer not to answer | | | | |
| Please specify: | _________________________________________________________ | | | | |
| Do you identify as transgender, gender diverse, and/or gender queer? | - Yes - No - Prefer not to answer | | | | |
| Do you identify as intersex? | - Yes - No - Prefer not to answer | | | | |
| What is your age? | _________________________________________________________ | | | | |
| What is your ethnicity? | - Hispanic/Latino - Not Hispanic/Latino - Prefer not to answer | | | | |
| What is your race (check all that apply)? | - American Indian or Alaskan Native - Hawaiian or Pacific Islander - Asian - Black or African American - White or Caucasian - Identify as:   __________________   - Prefer not to answer | | | | |
| Please specify: | _________________________________________________________ | | | | |
| What is your sexual orientation? | - Asexual - Bisexual - Lesbian - Gay - Heterosexual - Queer - Not Sure/Questioning - Identify as:   ___________________________________   - Prefer not to answer | | | | |
| Please specify: | _________________________________________________________ | | | | |
| What is the highest level of education you completed? | - Some college - Associate’s degree - Bachelor’s degree - Master’s degree - Doctoral degree (PhD, MD, JD, DrPH) - Other professional degree - Prefer not to answer | | | | |
| In what year did you complete your highest level of education? | _________________________________________________________ | | | | |
| At which type of institution do you work? | - University Hospital - Community/Private Hospital - Private Practice - Other: _____________________________ | | | | |
| Please specify: | _________________________________________________________ | | | | |
| What are your personal political leanings? | - Very Conservative - Conservative - Somewhat Conservative - Centrist/Moderate - Somewhat Liberal - Liberal - Very Liberal - Not political - Some other viewpoint - Prefer not to answer | | | | |
| Do you have any lesbian, gay, bisexual, transgender, gender diverse, queer or intersex family members? | - Yes - No - Prefer not to answer | | | | |
| Do you have any lesbian, gay, bisexual, transgender, gender diverse, queer, or intersex friends or coworkers? | - Yes - No - Prefer not to answer | | | | |
| Have you received cancer-specific training on the needs of sexual and gender minority patients with cancer? | - Yes - No - Prefer not to answer | | | | |
| How many hours of cancer-specific training on sexual and gender minority patient needs have you received?" | _________________________________________________________ | | | | |
| Would you like to receive training on the care of LGBTQI patients with cancer? | - Yes - No - Prefer not to answer | | | | |
| Which of the following cancer-specific topics focusing on sexual and gender minorities would you be interested in learning more about: | - Hormone-mediated tumors in transgender patients with cancer - End-of-life issues - Experienced health care disparities - Evidenced-based scientific issues - Policy and advocacy issues - Creating a welcoming environment - How to take a culturally competent history/intake - Additional suggestions:_____________________ | | | | |
| Please provide additional topic suggestion(s): | _________________________________________________________ | | | | |
| In what format would you like to receive training on the care of LGBTQI patients with cancer? | - Online learning modules - In-person training - Journal article - Other: ______________________________ | | | | |
| Please specify: | _________________________________________________________ | | | | |
